# Thymus Atrophy and Double-Positive Escape Are Common Features in Infectious Diseases

**DOI:** 10.1155/2012/574020

**Published:** 2012-02-01

**Authors:** Juliana de Meis, Désio Aurélio Farias-de-Oliveira, Pedro H. Nunes Panzenhagen, Naiara Maran, Déa Maria Serra Villa-Verde, Alexandre Morrot, Wilson Savino

**Affiliations:** ^1^Laboratory on Thymus Research, Oswaldo Cruz Institute, Oswaldo Cruz Foundation, Avenue Brasil 4365, Manguinhos, 21045-900 Rio de Janeiro, RJ, Brazil; ^2^Department of Immunology, Microbiology Institute, Federal University of Rio de Janeiro, 21941-590 Rio de Janeiro, RJ, Brazil

## Abstract

The thymus is a primary lymphoid organ in which bone marrow-derived T-cell precursors undergo differentiation, leading to migration of positively selected thymocytes to the T-cell-dependent areas of secondary lymphoid organs. This organ can undergo atrophy, caused by several endogenous and exogenous factors such as ageing, hormone fluctuations, and infectious agents. This paper will focus on emerging data on the thymic atrophy caused by infectious agents. We present data on the dynamics of thymus lymphocytes during acute *Trypanosoma cruzi* infection, showing that the resulting thymus atrophy comprises the abnormal release of thymic-derived T cells and may have an impact on host immune response.

## 1. Introduction

**Figure 1:**
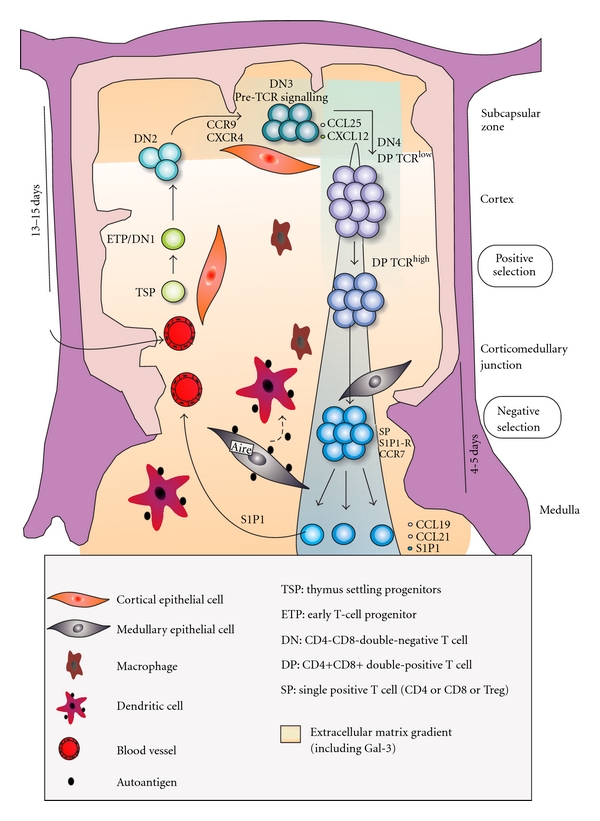
*Intrathymic differentiation of T cells*. Lymphocyte differentiation initiates when T-cell precursors enter the thymus through postcapillary venules located at corticomedullary junction. After entering the organ, cells interact with the thymic microenvironment (thymic epithelial cells, macrophages, dendritic cells, and fibroblasts), which ultimately lead to their proliferation and TCR rearrangement. Interactions between thymocytes and specialized thymic microenvironmental cells support and direct T cell differentiation by means of a series of interactions including receptor/coreceptor interactions (MHC-TCR, Integrin/ECM Proteins), cytokines (IL-1, IL-2, IL-3, IL-6, IL-7, IL-8, IFN-gamma), chemokines (as CCL25, CXCL12, CCL21), and hormones, with corresponding receptors. At the subcapsular zone, these thymocytes undergo TCR beta chain rearrangement and selection. Double-positive thymocytes migrate through the cortex and initiate TCR testing (positive selection). Positively selected thymocytes, located at the medulla, are screened for self-reactivity through negative selection. Residence in the medulla is followed by emigration, which is regulated by sphingosine-1-phosphate and its receptor (S1P1). Adapted from [1].

 The thymus is a primary lymphoid organ in which bone marrow-derived T-cell precursors undergo differentiation, leading to migration of positively selected thymocytes to the T-cell-dependent areas of secondary lymphoid organs [2]. Interactions between thymocytes and specialized thymic microenvironmental cells (thymic epithelial cells, macrophages, dendritic cells, and fibroblasts) support and drive T-cell differentiation from bone marrow-derived precursors, by means of a series of interactions including receptor/coreceptor interactions, cytokines, chemokines, and hormones [3–7], as illustrated in Figure 1.

 Thymopoiesis starts at the time that a T-cell precursor enters the thymus and interacts with local microenvironmental cells, which ultimately lead to their proliferation and further differentiation to the T-cell lineage. Various types of interactions take place, including those mediated by the class I and class II major histocompatibility complexes (MHC) expressed by microenvironmental cells, extracellular matrix proteins (ECM) such as laminin, fibronectin, and collagen, chemokines (as CCL25, CXCL12, CCL21), lectins such as galectin-3, various typical cytokines (IL-1, IL-2, IL-3, IL-6, IL-7, IL-8, IFN-gamma, and others), sphingosin-1-phosphate (S1P1), and hormones (thymulin, thymopoietin, thymosin-a1) [2, 5, 8–13]. T-cell differentiation depends on T-cell receptor (TCR) gene rearrangement and membrane interaction with MHC molecules.

The mechanisms by which progenitors home to the thymus have been suggested to be similar to those used by leukocytes to enter lymph nodes (selectins, chemokines receptors, and integrins) [1, 14, 15]. As soon as these thymic settling progenitors (TSP) enter the thymus close to the cortico-medullary junction, they generate early T-cell progenitors (ETP) or double-negative DN1 thymocytes, known to be CD117/c-KIT^+^, CD44^+^ CD25^−^ [16]. ETP or DN1 thymocytes evolve to DN2 and DN3 thymocytes that migrate to the subcapsular zone of the thymic lobules, where they rearrange the genes encoding the TCR beta chain, express pre-TCR receptor, and proliferate.

At the DN3 stage, the CXCL12/CXCR4 interaction contributes thymocyte proliferation and differentiation towards the DN4 and subsequently CD4^+^CD8^+^ (DP) stage [1, 17]. Double-negative thymocytes, TCR^−^CD4^−^CD8^−^, represent 5% of total thymocytes. Maturation progresses with the definite acquisition of TCR, CD4, and CD8 expression generating DP; double cells, which constitute 75–80% of the whole thymocyte population. Thymocytes that do not undergo a productive TCR gene rearrangement die by apoptosis, whereas those expressing productive TCRs interact with peptides presented by molecules of the major histocompatibility complex (MHC), expressed on microenvironmental cells. The result of this interaction determines the fate of thymocytes [2, 9, 18]. The positively selected thymocytes will escape from apoptosis and become mature CD4^+^ or CD8^+^ single-positive (SP) T cells (Figure 1). This is a highly rigorous process, and only a small proportion of the double-positive population survives [19]. Positive selection also results in lineage commitment so that the lymphocytes can be committed to either the CD4 or CD8 single-positive phenotype, depending on the class of MHC molecule with which the TCR interacts.

Intrathymic negative selection is essential to establish self-tolerance in the T-cell repertoire, deleting high-avidity TCR signaling thymocytes reacting to self-peptides presented by microenvironmental cells [2, 11, 18, 20].

Interestingly, along with CD4^+^ T-cell differentiation, two distinct groups of cells, with opposite roles, have been reported: the classical CD4^+^ T helper cells (cells that are able to trigger and/or enhance an immune response in the periphery) and regulatory CD4^+^CD25^+^FOXP3^+^ T cells, which are able to impair a given immune response [9, 21].

The data summarized above clearly demonstrate that the thymus is vital for the homeostatic maintenance of peripheral immune system, maturing both effector and regulatory T cells (Figure 1).

It has been well documented that the thymus undergoes an age-related atrophy [22]. Under normal circumstances, the decline in thymic cellularity in healthy subjects promotes minimal consequence. Nevertheless, over time, reduced efficacy of the immune system with age increases the rise of opportunistic infections, autoimmunity, and cancer [22–24].

In this paper, we present emerging data regarding accelerated thymus atrophy caused by infected agents and possible impact of this thymic atrophy to the host immune response. Moreover, we show that thymic-derived T cells are involved in the dynamics of lymphocyte populations in secondary lymphoid organs during acute *Trypanosoma cruzi *infection.

## 2. Parasite Infection Promotes Thymic Atrophy with CD4^+^CD8^+^ Thymocyte Depletion

As mentioned above, the thymus senses several exogenous agents, responding with atrophy, promoted by viruses (HIV, rabies virus), parasites (*Trypanosoma cruzi*, *Plasmodium berghei*, *Schistosoma mansoni, *and *Trichinella spiralis*), and fungi (*Paracoccidioides brasiliensis *and *Histoplasma capsulatum*) [9, 22, 25–40]. The mechanisms involved in the thymic atrophy in infectious disease are not completely elucidated and may vary. Nevertheless, common histological features occur, including decrease of cortical thymocytes and loss of clear-cut distinction in the corticomedullary region [9, 38, 41–47]. At least in some cases, such atrophy may be transient: biphasic reactions of the thymic cortex, characterized by initial atrophy and further restoration, were reported in experimental infections by *Histoplasma capsulatum *and *Toxoplasma gondii *[48, 49].

**Figure 2:**
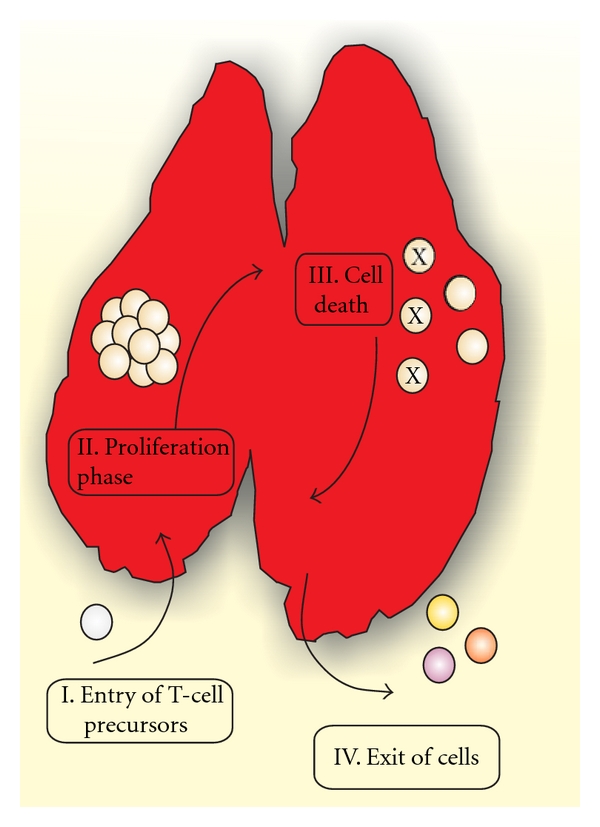
Possible mechanisms involved in thymic atrophy. I. Decreased number of precursor cells migrating into the thymus, II. Lower capacity in thymocyte proliferation during T-cell differentiation, III. Increased thymocyte death, and/or IV. Exit of immature T cells to peripheral tissues.

Thymic atrophy in infectious disease may reflect distinct nonmutually excluding events: decreased number of precursor cell entry into the thymus, lower capacity in thymocyte proliferation, increased thymocyte death, and/or increased exit of thymocytes to peripheral lymphoid tissues (Figure 2).

Although the migratory capacity of T-cell precursors to colonize the thymus in infectious disease remains unknown, data from the literature suggest that parasite-induced thymus atrophy comprises changes in involvement of proliferation, death, and exit of thymocytes.

## 3. Impaired Thymocyte Proliferation in *T. cruzi*-Infected Mice

It has been shown that mitogenic responses of thymocytes from *T. cruzi *acutely infected mice are reduced due to decrease in interleukin (IL)-2 production, which in turn is associated with high levels of IL-10 and interferon-*γ* [50]. It has also been suggested that changes in thymocyte subset proportions induced by *T. spiralis *infection are reflected in a reduced capacity of thymocytes to respond to the T-cell mitogen concanavalin A [45]. In contrast, thymocytes from *S. mansoni-*infected mice apparently exhibit similar concanavalin A-induced proliferative response, as compared to controls [38]. Conjointly, these data suggest that some (but not all) parasites induce decrease in the ability of thymocytes to proliferate, which in turn account for the resulting thymic atrophy.

## 4. Thymocyte Apoptosis Is a Common Feature in Acute Parasite Infections

 In the vast majority of infectious diseases coursing with thymic atrophy, the major biological event associated with thymocyte loss is cell death by apoptosis, as seem, for example, in experimental models of *Trypanosoma cruzi *and *Plasmodium berghei *infection [9]. Although CD4^+^CD8^+^ thymocytes are the main target population in infection, other subsets as DN and SP cells also depleted in infected thymus [30, 32, 42, 63, 64].

Glucocorticoid hormones are strong candidates to promote thymic atrophy and thymocyte death in parasitic infections. Serum glucocorticoid levels are upregulated in acute infections and promote DP thymocyte apoptosis through caspase-8 and caspase-9 activation [9, 56, 57, 65, 66] (Box 1). Such rise in serum glucocorticoids has been reported in experimental parasitic diseases such as malaria, American tripanosomiases or Chagas disease, African trypanosomiases or sleeping sickness, toxoplasmosis, leishmaniasis, and schistosomiasis [51, 56, 67–72]. In experimental acute *T. cruzi* infection, thymic atrophy and thymocyte depletion have been associated with both TNF and glucocorticoid serum levels [44, 65, 73].

Nevertheless, at least in *T. cruzi *infection, various and different biological mechanisms seem to be involved. *T. cruzi*-derived transsialidase, as well as host-derived galectin-3, extracellular ATP, and androgens have been pointed out as candidate molecules to enhance thymocyte death [44, 64, 69, 74–77]. Conversely, typical cytotoxic molecules such as Fas and perforin are not involved in thymus atrophy in *T. cruzi *infection [78].

## 5. Acute Infection Can Promote Abnormal Escape of Immature Thymocytes to the Periphery

T-lymphocyte migration is controlled by several molecular ligand/receptor interactions, including those involving ECM proteins, chemokines, and lectins [12, 13, 79–82].

In the thymus of mice acutely infected by *T. cruzi *or *P. berghei *alterations in expression of ECM proteins, chemokines, and/or galectin-3 have been described [5, 63, 64, 79, 83], which is in keeping with the abnormal appearance of thymus-derived immature DP lymphocytes in peripheral lymphoid organs and blood from infected hosts. These findings suggest that the premature scape of immature cells from the organ also contributes to the establishment of the thymic atrophy [38, 42, 84, 85]. Accordingly, it has been shown that thymocytes from *T. cruzi *acutely infected mice exhibited increased migratory responses to fibronectin and that abnormally high numbers of DP T cells migrate from the thymus to peripheral lymphoid organs. [42, 64, 83–86] (Box 2). Studies performed in experimental *P. berghei *infection have also demonstrated increased expression of ECM proteins, CXCL12 chemokine production, and enhanced migratory response of thymocytes from infected mice, when compared to controls [87].

## 6. Thymic Changes May Impact on the Immune Response of Infected Animals

**Figure 3:**
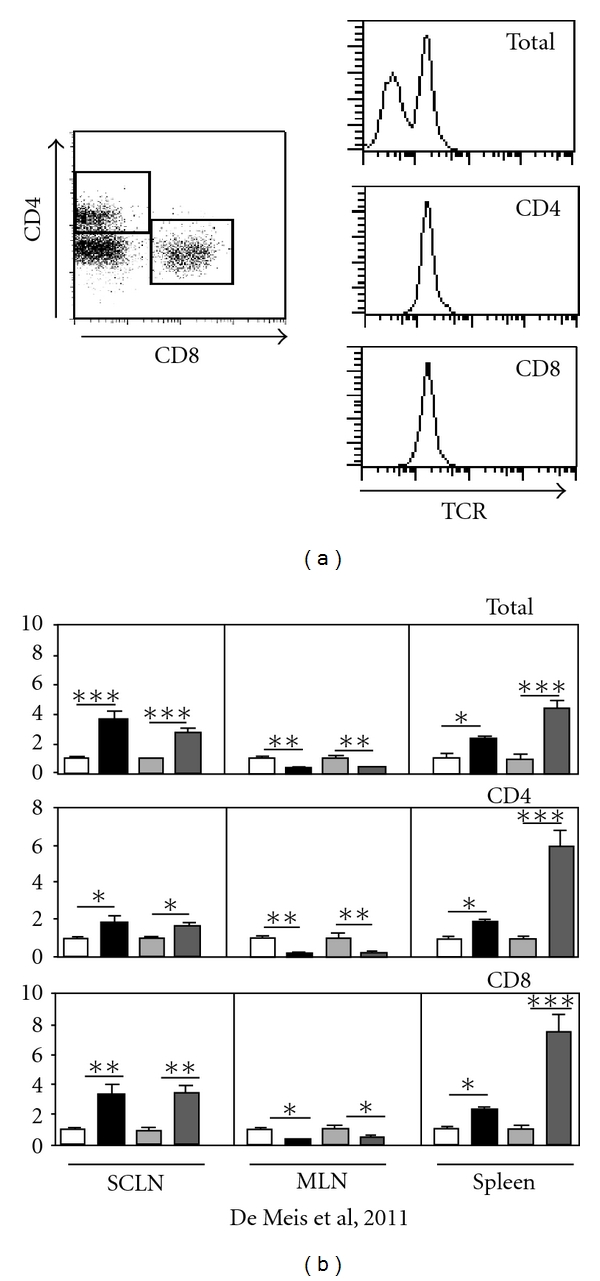
Thymectomy modulates splenic cell numbers during acute *Trypanosoma cruzi *infection. Mice were thymectomized and, six days later, were infected intraperitoneally by the Tulahuén strain of *T. cruzi*. Animals were killed at 19 days postinfection, and subcutaneous (SCLN), mesenteric (MLN), lymph nodes and spleen cell numbers were evaluated. (a) Representative data demonstrating TCR expression in CD4 and CD8 T cells in SCLN, MLN, and spleen, analyzed by flow cytometry. (b) Data show fold change of 6–8 animals/group where (white rectangle) represents sham-operated control, (black rectangle) sham-operated infected, (light grey rectangle) thymectomized control, and (dark grey rectangle) thymectomized infected mice. Results were representative of three different experiments and were expressed as mean ± standard deviation, ns: not significant, *P < 0.05, **P < 0.01, and ***P < 0.001, after comparison by One Way ANOVA.

Acute *T. cruzi *infection in mice leads to strong activation of innate and adaptive immune responses. Splenomegaly and expansion in subcutaneous lymph nodes (SCLN) were reported, mediated by persistent T- and B-cell polyclonal activation [63, 88–91]. Conversely, atrophy in thymus and mesenteric lymph nodes (MLN) has been observed along with infection [9, 43, 92]. We have previously demonstrated that MLN atrophy in *T. cruzi* infection mice was associated with massive lymphocyte apoptosis, mediated by TNF, Fas, and caspase-9 [63, 88, 92]. The role of thymus-derived T cells in secondary lymphoid organ dynamics remains unclear. In order to analyze the role of the thymus upon regional immune response in secondary lymphoid organs from acute *T. cruzi *infected mice, thymectomized male BALB/c mice or sham-operated counterparts were infected with 100 blood-derived trypomastigotes from Tulahuén strain of *T. cruzi*. In the peak of parasitemia (18–21 d.p.i), mice were killed, and subcutaneous, mesenteric lymph nodes as well as spleen were analyzed. As demonstrated in Figure 3, thymectomy in noninfected mice does not alter lymphocyte counts in the spleen, SCLN, and MLN. However, absence of thymic-derived T cells during acute infection increased the number of splenocytes (Figure 3). In this respect, it has been demonstrated that thymus-derived *γδ*TCR^+^ T cells removed from the spleen exhibit suppressor activity for T lymphocytes [93]. Moreover, as showed in thymectomized *T. cruzi *chronically infected animals, thymic removal may act by downregulating immunoregulatory mechanisms, leading to an exacerbation of autoimmune reactions believed to be involved in the generation of myocardial damage [94].

 Interestingly, no changes were observed in SCLN cell expansion and MLN atrophy between infected sham and thymectomized mice, suggesting that suppressor T cells migrate preferentially to the spleen (Figure 3). All together, these data indicates that thymic-derived T cells can exert immunoregulatory in the spleen during acute *T. cruzi* infection.

## 7. Conclusion

Several pathogens, including *T. cruzi*, cause thymic atrophy. Although the precise mechanisms underlying this phenomenon are not completely elucidated, most likely it is linked to a particular pathogen-host relationship. Recently, we addressed whether the changes of the thymic microenvironment promoted by an infectious pathogen would also lead to an altered intrathymic negative selection of the T-cell repertoire. By using a *T. cruzi *acute infection model, we have seen that, despite the alterations observed in the cortex and medullary compartments undergoing a severe atrophy during the acute phase, the changes promoted by the infection in the thymic architecture do not affect the negative selection.

Although the intrathymic checkpoints necessary to avoid the maturation of T cells expressing potentially autoreactive “*forbidden*” T-cell receptors are present in the acute phase of murine Chagas disease, circulating CD4^+^CD8^+^ T cells have been reported in humans as well as in animals such as mice, chicken, swine, and monkeys [9, 62, 85]. The existence of this unconventional and rare lymphocyte population in the periphery was explained as a premature release of DP cells from the thymus into the periphery, where their maturation into functionally competent single-positive cells continues.

Most importantly, there is considerable evidence of an increased frequency of peripheral CD4^+^CD8^+^ T cells not only during acute *T. cruzi *infection but also in viral infections. For example, in human immunodeficiency virus or Epstein-Barr virus infections, the percentage of DP cells can increase to 20% of all circulating lymphocytes [95–97]. This fluctuation is also present in the secondary lymph nodes as we demonstrated in the experimental model of Chagas disease, in which DP-cell subset increases up to 16 times in subcutaneous lymph nodes [83, 85]. During the course of infection, these peripheral DP cells acquire an activated phenotype similar to what is described for activated and memory single-positive T cells with high IFN-*γ* production, CD44^+^CD69^+^ expression, and cytotoxic activity [62].

Furthermore, similar to previous studies showing high cytotoxic activity and effector memory phenotype of extrathymic DP cells in *cynomolgus *monkeys and in a chimpanzee experimental infection with hepatitis C virus [95], our results indicate that the DP cells purified from peripheral lymphoid tissues of chagasic animals show cytotoxic activity as compared to naïve single-positive CD4^+^ or CD8^+^ T cells.

Most likely, the presence of peripheral, mature, and activated DP lymphocytes challenges the perception of the T-cell populations involved in adaptive immune responses during the infection. The presence of peripheral activated DP cells with potentially autoreactive TCR may contribute to the immunopathological events possible related to several pathogen infections. In the Chagas disease model, we have demonstrated that increased percentages of peripheral blood subset of DP cells exhibiting an activated HLA-DR^+^ phenotype are associated with severe cardiac forms of human chronic Chagas disease [62]. The role of these HLA-DR^+^ DP T cells in myocardial damage and host pathologies is unknown. However, correlations between the changes in the numbers of DP T-cell subsets and the extent of inflammatory lesions may represent a clinical marker of disease progression in parasitic infections and may help the design of novel therapeutic approaches for controlling infectious diseases.

## Figures and Tables

**Box 1:**
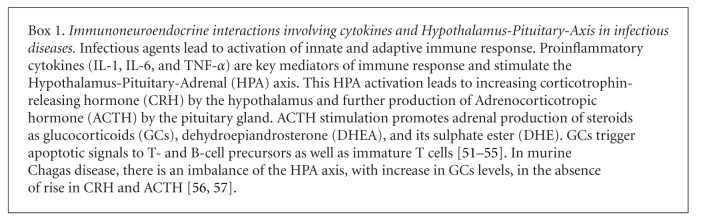


**Box 2:**
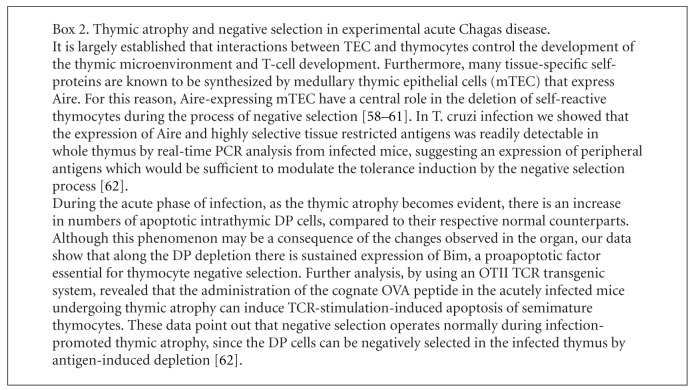


## Abbreviations

*T. cruzi:*: *Trypanosoma cruzi*
DP T cells: CD4^+^CD8^+^ double-positive T cells
AIRE: Autoimmune regulator gene
TRAs: Tissue-restricted antigens
TCR: T cell receptor
TEC: Thymic epithelial cells.
